# Supplementary material for: Comprehensive live-cell imaging analysis of cryptotanshinone and synergistic drug-screening effects in various human and canine cancer cell lines
Source: PLoS One. 2021 Feb 5;16(2):e0236074. doi: 10.1371/journal.pone.0236074 (PMC7864433; doi:10.1371/journal.pone.0236074)
Supplement: Attachment — Submitted filename: PONE reviewer comments bittner .docx [file pone.0236074.s001.docx]

PONE-D-20-19784
Comprehensive Live-cell Imaging Analysis of Cryptotanshinone and Synergistic Drug-Screening Effects in Various Human and Canine Cancer Cell Lines
PLOS ONE

Dear Chakrabhavi Dhananjaya Mohan, Ph.D,

We thank you very much for the opportunity to revise this manuscript and the reviewers’ thoughtful comments. We have attempted to address all of the comments as outlined below in the updated manuscript. A response to each review is included below in italics.

Reviewer #1: The research article entitled “ Comprehensive Live-cell Imaging Analysis of Cryptotanshinone and Synergistic Drug2 Screening Effects in Various Human and Canine Cancer Cell Lines” by Bittner etal, have described the exposure of varied drug combination against human and canine cancer cells and highlighted the results of CT efficacy at combating cancer using comprehensive live-cell drug-screening analysis. The experiments are well designed and detail investigation/interpretations of the results have been carried out from the authors. The results are statistically validated and are in the interest of researchers with significant data which will be useful for cancer therapeutics. I feel the article can be accepted for publication. However minor spelling mistakes have been noticed which can be fixed by the authors.

*The manuscript has been reviewed and any typos that we identified have been fixed.*

Reviewer #2: This study reported the anticancer activity of cryptotanshinone against a number of human and canine cancer cell lines using a comprehensive live-cell imaging analysis. These findings are interesting, and provide useful information regarding the potential application of this natural product in treating cancer. The following concerns need to be addressed.

1. The authors may consider including several normal cells in the imaging analysis in order to show the selective cytotoxicity of cryptotanshinone.

*Though normal cell lines were not tested in this study, we have provided the following references in the introduction as well as some additional language that support the use of this drug against cancer cell lines while sparing normal/healthy cell lines.*

2. Since this is a cell imaging analysis, it would be better to show some representative images of cells after treatment with cryptotanshinone, in particular a time-dependent response.

*An additional figure (figure 5) has been added to the manuscript to show the effects of CT on the canine OS cell lines UWOS2 over 12 hours.*

3. Regarding the anticancer activity of drug combination with cryptotanshinone, the potential application of cryptotanshinone in reversing multidrug resistance of cancer cells needs to be discussed.

*A paragraph discussing this has been added to the discussion.*

4. A detailed method regarding quantitative analysis of the images needs to be described.

*The following reference and more detail have been added to the methods section describing in detail the quantitative analysis of the images.*

*Hua, Jianping, Chao Sima, Milana Cypert, Gerald Gooden, Sonsoles Shack, Lalitamba Alla, Edward Smith, Jeffrey M. Trent, Edward R. Dougherty, and Michael L. Bittner. "Tracking transcriptional activities with high-content epifluorescent imaging." Journal of biomedical optics 17, no. 4 (2012): 046008.*

Sincerely,

Heather Wilson-Robles, DVM
